# Chronic Urethritis of Gonococcic Origin

**Published:** 1901-08

**Authors:** 


Chronic Urethritis of Gonococcic Origin. By J. De Keersmaecker, Chief of Service, Diseases of the Urinary Organs at the Centralklinik of Antwerp; and J. Verhoogen, Agrégé at the University of Brussels; Chief of Service, Diseases of the Urinary Organs, at the Polyclinique Libre. Translated and edited with notes by Ludwig Weiss, M. D., Attending Physician to the Genito-urinary and Skin Service, German Poliklinik; Dermatologist to the Hebrew Orphan Asylum, New York, etc. Small 8vo, pp. 274. New York: William Wood & Co. 1901.

This volume appears for the first time in the English language. It has been translated, edited, and, it might be said, revised by Weiss. It is well that it has been revised, for although the German authors have produced a very interesting and scientific volume, there would have been so much lacking that it is a question whether the work would have been given the standing it deserved by the American medical profession, except for the editor's note.

It is pleasing to read Weiss's advice as to the use of urethral antisepsis and pre-urethral cleansing before the passage of instruments of any kind, yet in Chapter XII. he says: "But in our opinion it is not essentially necessary in cases where a noninfectious process is to be combatted." This should have been omitted for the cases are so few in which instrumentation is necessary when there has been no previous trouble of an inflammatory nature, that it is safe to advise antisepsis in every instrumental exploration of the urethra.

Weiss admits that chills do occur even after urethral flushing with a 4 per cent. boracic acid solution. The reviewer thinks that if this were used full strength or a formaldehyde solution 1-1000, chills would never occur, and, furthermore, that neither of these solutions would in any way injure the delicate epithelial lining.

Chapter XIII., devoted to mechanical treatment, advises the use of dilators instead of sounds in promoting the absorption of inflammatory patches and the like. Neither authors nor translator mention one word as to preparation of the canal before these instruments are used. They do admit, however, that in nearly every case following dilatation there is an increase of discharge, at times to an extent where this form of treatment must be discontinued and resource given to astringents.

It is a fact that a sound, and especially an instrument covered with rubber, will cause more or less irritation even in a healthy canal. What then must be the result when such an instrument is passed through a canal suffering at the time or only recovering from inflammatory action? It is the reviewer's opinion that these exacerbations following instrumentation could be eliminated, providing the membrane were brought to as near a normal condition as possible before dilatation was instituted. Exacerbations not only could be obviated, but a cure could be recorded in much less time than is shown by some of the cases reported.

In Chapter XV., added by Weiss, that great question of gonorrhea and marriage is well considered, much space being given to the cocci as an infecting agent. He also speaks of pus cocci, but does not give them the space they deserve. It is an assured fact that no practitioner would allow a patient to marry while gonococci were present, but would it be advisable to allow marriage to take place even though repeated examination would show their absence, if the urine contained pus, known to come from the lower urinary region and this on careful examination showing strepto- or staphylococci? It is true they could not cause specific (gonorrhea) involvement, but they would undoubtedly produce inflammation, which would run a course just as severe as though gonococci were present. Of course, this question is a grave one for consideration and should only be dealt with by a liberal, yet well-informed microscopical operative.

Several beautiful plates are introduced showing the urethra as seen through an endoscopic tube, but it must be conscientiously said that the only way to learn the urethra is by seeing it in the living subject, under the instruction of an experienced man.

